# Bachelor completion and dropout rates of selected, rejected and lottery-admitted medical students in the Netherlands

**DOI:** 10.1186/s12909-019-1511-4

**Published:** 2019-03-12

**Authors:** Catharina M. P. Vos, Anouk Wouters, Marianne Jonker, Marian de Haan, Marleen A. Westerhof, Gerda Croiset, Rashmi A. Kusurkar

**Affiliations:** 1 Department of Research in Education, Amsterdam UMC, Vrije Universiteit Amsterdam, VUmc School of Medical Sciences, Amsterdam, the Netherlands; 2 LEARN! research institute for learning and education. Faculty of Psychology and Education, VU University, Amsterdam, the Netherlands; 3 Department of Policy, Innovation and Quality, Amsterdam UMC, Vrije Universiteit Amsterdam, VUmc School of Medical Sciences , Amsterdam, the Netherlands; 4 At the time of the study: Department of Epidemiology & Biostatistics, VU University Medical Center, Amsterdam, the Netherlands; 5 Present address:Department of Health Evidence, Radboud University Nijmegen, Medical Center, Nijmegen, the Netherlands; 6 UMC Groningen, Groningen, the Netherlands

**Keywords:** Admissions, Selection, Lottery, Medical school

## Abstract

**Background:**

Evidence for the effectiveness of the selection of medical students is weak. This study aimed to examine the added value of a two-step selection procedure (first step non-academic, second step academic tests) to a pre-university GPA-based lottery procedure. Because previous research has suggested that participation in selection (regardless of the outcome) is a predictor of study success, this study is the first to include students who initially applied for selection, then refrained from (actively) participating in selection and were eventually admitted through lottery.

**Methods:**

Bachelor completion and dropout rates of selected (*n* = 416) and lottery-admitted students from four cohorts (2006–2009) were compared using logistic regression analysis. Four groups of lottery-admitted students were distinguished: students who were rejected after step 2 (*n* = 57), were rejected after step 1 (*n* = 169), withdrew during selection step 1 (*n* = 42) and students who only applied for lottery (*n* = 366). Covariates included gender, age, pre-university GPA and cohort.

**Results:**

There was a significant association between admission group and obtaining a bachelor degree in three years. Selected students were more likely to obtain a bachelor degree within three years (64.2% versus 51.6%; OR = 1.7) or four years (81.5% versus 74.3%; OR = 1.6) than students who only applied to a lottery (*p* <  0.05); selected students also seemed more likely to obtain all Year-1 course credits than students who withdrew during step 1 (40.4% versus 21.4%; OR = 2.3; *p* <  0.05). We found no significant association between dropout and admission groups. Students rejected at step 1 or 2 did not perform significantly different from selected students on any of the outcome measures.

**Conclusions:**

The findings indicated that students at risk for study delay in the preclinical phase in our context were more likely to refrain from applying to a demanding selection procedure when a less demanding alternative was available. We found no significant associations between the non-academic and academic selection steps and bachelor completion and dropout rates. These findings suggest that the presence of the selection was more important than these specific selection components. In follow-up research, we plan to investigate the associations between the admission groups and outcome measures in the clinical phase.

## Background

**Fig. 1:**
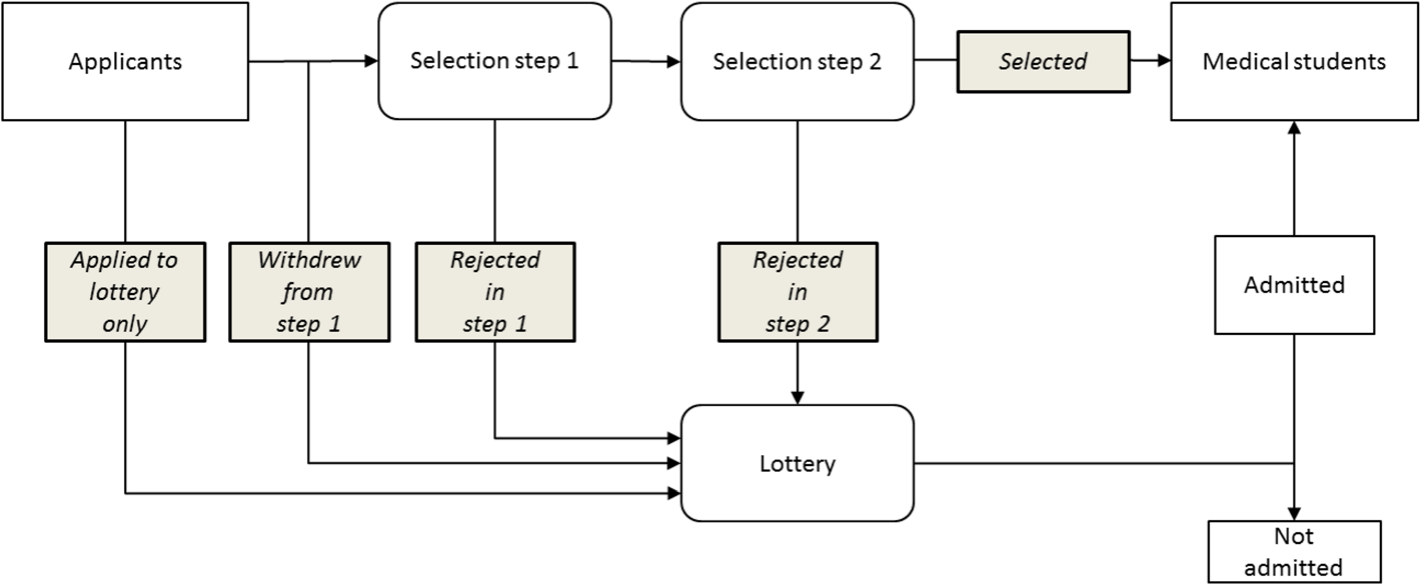
Flowchart of admissions

Evidence for the effectiveness of the selection of students for medical study is weak [1, 2]. This is because students who are rejected in selection generally do not enrol in medical study. Therefore, it remains unclear how these students would perform in medical study compared to those who were selected. The previous Dutch admission system did provide the opportunity to conduct such comparisons. In the present study, these comparisons were conducted for selected students, rejected students (in different selection steps) who had subsequently been admitted through a lottery procedure, students who had withdrawn from participation in selection, and students who only applied to the lottery procedure (see Fig. 1). This allowed for the investigation of the added value of a two-step selection procedure to a random selection through weighted lottery. It also allowed for the further exploration of the previously suggested participation effect of selection, which suggests that students who voluntarily participate in a qualitative selection procedure outperform those who only opt for a lottery procedure [3, 4].

Various selection tools have been studied, and research has yielded strong evidence for the predictive validity of previous academic performance for future academic performance. Evidence for the reliability and validity of other, mainly non-academic tools remains weak [1, 5]. An issue of concern is that conclusions are mainly drawn from research assessing the value of selection tools among only students who have been successful in the selection procedure. Usually, data on students who were unsuccessful in selection are not available.

For years, students in the Netherlands who were rejected in selection could be admitted to medical study due to a unique admissions system. Applicants who obtained a pre-university grade point average (pu-GPA) of ≥8 on a scale of 1 to 10 (*top GPA*) were granted direct admission to medical study. Those who finished high school with a GPA below 8 could participate in a qualitative selection procedure (*selection*) and/or enrol in a weighted lottery, in which the chances of admission increased with a higher GPA (*lottery*). When rejected in selection, applicants were automatically enrolled in the lottery procedure [6]. Previous research studying the three admission groups in this setting has yielded inconsistent findings. Top GPA students consistently outperform other students in academic and non-academic performance outcomes [3, 4, 7–10], while comparisons between selected students and lottery-admitted students on various study performance outcomes yield either no statistically significant differences or show that selected students perform significantly better than lottery-admitted students [3, 4, 8–15]. A Danish study also found that students who were admitted based on a qualitative selection procedure outperformed students admitted based only on their pu-GPA [16].

Differences between the performances of selected and lottery-admitted students are generally small. The fact that the lottery-admitted group consists of two distinct types of students may explain this. The first group only applies for the lottery procedure, which requires negligible effort, while the second group enrols through the lottery after they have put time and effort into participating in selection, but were nonetheless rejected. This has led some scholars to examine whether students participate in selection before being admitted through lottery as well as after which selection step students are rejected. Participation in a demanding selection procedure seems to predict better medical school performance [3, 4, 10, 15–17]. An explanation for this was attributed to better motivation among students who put effort into selection participation, but this attribution has not been substantiated by research [10]. Moreover, performance differences were not always found [3, 4, 10, 15].

Research on a selection procedure consisting of a non-academic first step using a portfolio and an academic second step involving cognitive tests has indicated that success in the academic step is related with lower dropout rates, while success in the non-academic step is related with higher clerkship grades [15]. In another comparative study, an academic selection procedure and a non-academic selection procedure were used simultaneously. Students selected through the non-academic selection procedure outperformed students selected through the academic selection procedure in clinical performance, while dropout rates were lower among the students selected through the academic selection procedure. Course grades did not differ between the two groups [18]. It seems that using a non-academic step alone is insufficient; instead it must be accompanied by an academic step [11, 18].

Students who withdrew from the selection procedure also enrolled in the lottery procedure. This group is interesting because applicants adopt a strategic approach when choosing which medical school they apply to, supposedly based on how they perceive their chances of success in the selection procedure [19]. Still, some applicants decide to withdraw from selection. To the best of our knowledge, this group has not been studied before. The aim of the current study was to gain insight into the value of non-academic and academic selection steps and to investigate whether a demanding selection procedure elicits a self-selection effect in which applicants who will be less successful in medical study are discouraged from applying. We compared bachelor completion and dropout rates for students admitted either via a two-step selection process (first step non-academic, second step academic tests) or by weighted lottery based on pu-GPA. Length of training (bachelor completion on time or within four years) was chosen as an outcome variable to enable comparisons with other studies comparing academic performance of selected and lottery-admitted students using bachelor completion as an outcome measure [11, 18, 20]. In so doing we thus focus on students’ ability to successfully complete the mainly knowledge-based phase of medical training, as the bachelor phase emphasizes academic and cognitive abilities rather than the more practical competencies of doctors such as problem-solving skills, people skills, and dealing with uncertainty. The two-step selection procedure was the same as that in previous research [11, 13–15]. However, the previous studies were carried out at another medical school. Moreover, in previous studies, selection was relatively new to the applicants, and lottery was still a habitual procedure; whereas in our research, more recent cohorts, comprising individuals who entered medical study when selection was becoming more common in the Netherlands, were investigated. The variation in the findings to date seems to imply that context matters. This study adds to the literature by investigating selection and lottery in a different context and by including a new group of students, i.e., students who withdrew from selection but were admitted through lottery. We hypothesised that if each selection step cumulatively contributes to identifying students who will perform best in the medical programme, then it can be expected that students who meet both academic and non-academic criteria and are successful in all selection steps are more likely to complete their medical bachelor degree (on time).

The primary research question was:

Do selected students in the preclinical phase of medical school more often obtain their bachelor degree within three years and drop out less often than students who

- were rejected in step 2 from the selection;
- were rejected in step 1 from the selection;
- withdrew from the selection procedure during step 1;
- or only participated in the lottery?

The secondary research question was:

Do selected students in the preclinical phase of medical school more often obtain 60 European Credits (EC) in Year-1 and their bachelor degree in four years than students who

- were rejected in step 2 from the selection;
- were rejected in step 1 from the selection;
- withdrew from the selection procedure during step 1;
- or only participated in the lottery?

## Methods

### Context

This study was performed at VUmc School of Medical Sciences, Amsterdam, the Netherlands. Each year, 350 students enrol in the medical programme. The (vertically integrated) medical curriculum consists of a three-year preclinical (bachelor) and a three-year clinical (master) phase [21]. This study pertains to the performance of students in the bachelor phase. In the first year, the programme consists of eight theoretical courses of three to five weeks with knowledge tests at the end, a two-week research methodology course, and a four-week internship as a nursing assistant. In the second year, the theoretical courses and tests are complemented with a five-day internship in general practice. The third year consists of five 6-week theoretical courses with knowledge tests and six weeks of science education. Assessments in the programme mostly consist of (cognitive) course-dependent tests but also include assessments of professional behaviour, skills exams and completion of obligatory practicals.

### Admission procedures

At the time of data collection, students could enter the undergraduate medical programme through direct access based on a top pu-GPA, lottery or selection (a maximum of 30% of the cohort was admitted through selection in 2006 and 2007; a maximum of 50% of the cohort was admitted through selection in 2008 and 2009). The selection procedure was the same two-step procedure as that used at Erasmus University in Rotterdam [13]. In short, step 1 consisted of scoring applicants’ portfolios containing their extracurricular activities (in health care, at the organisational level or excelling in sports or arts) and a statement on motivation. Applicants who met the threshold score for step 1 qualified for participation in step 2. In step 2, applicants were provided study materials for self-study and attended an on-campus lecture. One week later, they took five multiple-choice tests: a lecture-based knowledge and insight test, a calculation test, an interpretation of scientific literature test, an spatial orientation anatomy test and either a philosophy listening test (2006 and 2007) or a logical reasoning test (2008 and 2009).

### Study sample

A total of 1401 students who were admitted to medical study at VUmc School of Medical Sciences in September 2006, 2007, 2008 and 2009 were included in the study. Students who were admitted based on their top GPA (*direct access*; 9%, *n* = 126) did not need to participate in the lottery or selection. Because this study focused on the added value of a two-step selection procedure to a lottery procedure, analyses were limited to the selected (33%, *n* = 461) and lottery-admitted groups (52%, *n* = 733). For 6% of the students (*n* = 81), their admission data were unavailable, or their admission was based on special circumstances. For 12% (*n* = 144) of selected and lottery-admitted students, their pu-GPA, an important confounder, was unavailable. These students were excluded from the present study. For the analyses, we distinguished between selected students (*n* = 416) and four groups of lottery-admitted students: students who only applied for lottery (*n* = 366), students who applied for selection but withdrew during step 1 (*n* = 42), students who were rejected after step 1 (*n* = 169), and students who were rejected after step 2 (*n* = 57). A further breakdown of the groups is presented in Table 1.

**Table 1:** Students’ gender, age at the beginning of medical study, cohort and pu-GPA by admission group

	Gender (n)		Cohort (n)				Age at start of studies (yrs)		Pu-GPA (1–10)^a^	
	M	F	2006	2007	2008	2009	Mean	SD	Mean	SD
Selected (*n* = 416)	149 (36%)	267 (64%)	83	78	132	123	20.0	1.8	7.0	0.4
Rejected in step 2 (*n* = 57)	14 (25%)	43 (75%)	5	16	25	11	19.4	1.4	7.1	0.5
Rejected in step 1 (*n* = 169)	43 (25%)	126 (75%)	48	49	34	38	19.2	1.0	7.0	0.5
Withdrew from step 1 (*n* = 42)	21 (50%)	21 (50%)	6	19	12	5	19.2	1.5	7.1	0.5
Applied to lottery only (*n* = 366)	139 (38%)	227 (62%)	115	99	70	82	19.2	1.2	7.1	0.5
*Total (n = 1050)*	366 (35%)	684 (65%)	257	261	273	259	19.5	1.5	7.1	0.4

^a^Passing grade for pre-university education is ≥5.5

### Outcome measures

We chose outcome measures that reflected success in the first three years of medical education, i.e., the bachelor programme, and that were in line with previous studies to enable comparisons [11, 18, 20]. Obtaining a bachelor degree (180 ECs) within three years (standard time to complete programme) and dropping out without a diploma up to the sixth year after admission were the main outcomes of interest in this study. *Dropout* in the present study was defined as having no registration as a student in the program and having no bachelor degree of Medicine. We further explored Year-1 completion (i.e., obtaining all 60 ECs) in one year and obtaining a bachelor degree (180 ECs) within four years as secondary outcome measures. Within the European Credit Transfer and Accumulation System, one EC represents 28 h of study workload.

### Statistical analyses

We carried out binary logistic regression analyses to test associations between the different outcome measures and admission steps. We checked for confounders for this association with a forward selection procedure as described by Twisk [22]. Possible confounders, i.e., variables that might influence the association between the admission group and the outcome measure, were gender, age at start of medical school, cohort (because changing legislation and admissions policies of other medical schools may have influenced both the applicant pool and the study outcomes in the timeframe differently for the cohorts and are therefore of interest) and pu-GPA (standardised by cohort), and their interactions (cohort*age, age*gender and gender*pu-GPA). For the primary outcome measures, we corrected for multiple comparisons by applying the Holm-Bonferroni method. We performed the analyses using SPSS 22.0.

### Ethical considerations

All personally identifying student data were removed before analysis. All data were collected for admission and study progress monitoring purposes. Therefore, student or applicant consent for use of the data was not required. This study was approved by the ethical review board of the Netherlands Association for Medical Education (NVMO-ERB, file number 921).

## Results

The performance outcomes of the admission groups and the results from the regression analyses are presented in Table 2 and described below.

**Table 2:** Associations between admission groups and bachelor completion and dropout rates unadjusted and adjusted for confounders

			Unadjusted for confounders					Adjusted for confounders				
	N	% of students	B	*p*	OR	CI min	CI max	B	*p*	OR	CI min	CI max
Primary outcome measures												
Bachelor degree in three years*				**< 0.001**					**< 0.001**			
Selected (*n* = 416)	267	64,2%	ref	ref	ref	ref	ref	ref	ref	ref	ref	ref
Rejected in step 2 (*n* = 57)	29	50,9%	−0,55	0.05	0,58	0,33	1,01	−0,7	0.02	0,5	0,28	0,90
Rejected in step 1 (*n* = 169)	97	57,4%	−0,29	0.13	0,75	0,52	1,08	−0,25	0.22	0,78	0,53	1,16
Withdrew from step 1 (*n* = 42)	18	42,9%	−0,87	**< 0.01**	0,42	0,22	0,80	−0,83	0.02	0,44	0,22	0,87
Applied to lottery only (*n* = 366)	189	51,6%	−0,52	**< 0.001**	0,6	0,45	0,79	−0,53	**< 0.001**	0,59	0,43	0,80
*Total (n = 1050)*	*600*	*57,1%*										
Dropout in 6th year**				0.20					0.07			
Selected (*n* = 416)	31	7,5%	ref	ref	ref	ref	ref	ref	ref	ref	ref	ref
Rejected in step 2 (*n* = 57)	2	3,5%	−0,8	0.29	2,21	0,52	9,51	−0,68	0.37	1,97	0,45	8,57
Rejected in step 1 (*n* = 169)	14	8,3%	0,12	0.73	0,89	0,46	1,72	0,27	0.44	0,76	0,38	1,52
Withdrew from step 1 (*n* = 42)	7	16,7%	0,91	0.05	0,4	0,17	0,98	1,14	0.02	0,32	0,13	0,81
Applied to lottery only (*n* = 366)	34	9,3%	0,24	0.35	0,79	0,47	1,31	0,48	0.08	0,62	0,36	1,06
*Total (n = 1050)*	*88*	*8,4%*										
Secondary outcome measures												
Year-1 completion in one year***				0.12					0.16			
Selected (*n* = 416)	168	40,4%	ref	ref	ref	ref	ref	ref	ref	ref	ref	ref
Rejected in step 2 (*n* = 57)	26	45,6%	0,21	0.45	1,24	0,71	2,16	0,21	0.49	1,23	0,69	2,20
Rejected in step 1 (*n* = 169)	63	37,3%	−0,13	0.49	0,88	0,61	1,27	−0,05	0.79	0,95	0,64	1,40
Withdrew from step 1 (*n* = 42)	9	21,4%	−0,91	**0.02**	0,4	0,19	0,86	−0,85	**0.04**	0,43	0,19	0,94
Applied to lottery only (*n* = 366)	153	41,8%	0,06	0.69	1,06	0,80	1,41	0,12	0.45	1,12	0,83	1,52
*Total (n = 1050)*	*419*	*39,9%*										
Bachelor degree in four years****				0.11					0.07			
Selected (*n* = 416)	339	81,5%	ref	ref	ref	ref	ref	ref	ref	ref	ref	ref
Rejected in step 2 (*n* = 57)	45	78,9%	−0,16	0.65	0,85	0,43	1,69	−0,38	0.31	0,69	0,33	1,42
Rejected in step 1 (*n* = 169)	136	80,5%	−0,07	0.78	0,94	0,59	1,47	−0,11	0.66	0,9	0,55	1,46
Withdrew from step 1 (*n* = 42)	30	71,4%	−0,57	0.12	0,57	0,28	1,16	−0,68	0.08	0,51	0,24	1,09
Applied to lottery only (*n* = 366)	272	74,3%	−0,42	**0.02**	0,66	0,47	0,92	−0,49	**0.01**	0,61	0,42	0,89
*Total (n = 1050)*	*822*	*78,3%*										

Significant differences at a *p* < 0.05 level are highlighted in bold text. Primary outcomes were tested using Holm-Bonferroni correction. Secondary outcomes were tested without correction for multiple comparisons because of the exploratory nature of these analyses

*Adjusted for pu-GPA, age and gender

**Adjusted for pu-GPA, age and cohort

*** Adjusted for pu-GPA, age, gender and gender*pu-GPA

****Adjusted for pu-GPA, age, gender and cohort

### Primary research outcomes

#### Obtaining a bachelor degree within three years

A significant association was found between the variables ‘obtaining a bachelor degree within three years’ and ‘admission group’ (*p* < 0.05). Selected students more often obtained their bachelor degree within three years than students who had withdrawn during step 1 (OR = 0.42, *p* < 0.01) and students who only applied for lottery (OR = 0.60, *p* < 0.01). After adjusting for the confounders pu-GPA, age and gender, the association between the outcome variable and the admissions group remained significant (*p* < 0.01). Differences remained significant for students who only applied for the lottery (OR = 0.59, *p* < 0.01). No other significant effects were found.

#### Dropout in 6th year

No significant association was found between the variables ‘dropout’ (no registration as a student in Year-6 without a bachelor degree) and ‘admission group’.

### Secondary research outcomes

#### Year-1 completed in one year

No overall significant association was found between the variables ‘Year-1 completed in one year’ and ‘admission group’, but selected students more often obtained all Year-1 ECs in one year than students who had withdrawn during step 1 (OR = 0.40, *p* < 0.05). After adjusting for the confounders pu-GPA, age, gender and the interaction between gender and pu-GPA, the association between the outcome variable and the admission group was not significant, but the difference between the selected group and the students who had withdrawn remained significant (OR = 0.43, *p* < 0.05). No other significant effects were found.

#### Obtaining a bachelor degree within four years

No overall significant association was found between the variables ‘obtaining a bachelor degree within four years’ and ‘admission group’, but selected students more often obtained their bachelor degree within four years than students who only applied for the lottery (OR = 0.66, *p* < 0.05). After adjusting for the confounders pu-GPA, age, gender and cohort, the association between the outcome variable and the admission group was not significant, but the difference between the selected group and the students who only applied for the lottery remained significant (OR = 0.61, *p* < 0.01). No other significant effects were found.

## Discussion

The aim of this study was to determine whether selected students were more likely to complete their medical courses (on time) compared to students who were rejected during the non-academic or academic selection step of the selection procedure, students who withdrew their selection participation, and students who only participated in the lottery procedure. We focused on early medical school performance, i.e., obtaining a bachelor degree in three or four years, dropout and Year-1 course completion in one year. After adjusting for confounders, we found a significant association between the selection step and bachelor completion in three years only. Post-hoc comparisons between the different admission groups revealed that selected students more often obtained a bachelor degree in three years than students who only applied for the lottery. Furthermore, the findings suggest that selected students may be more likely to obtain their bachelor degree in four years than students who only apply to the lottery and more likely to complete their Year-1 courses in one year than students who withdraw from the selection procedure during the first, non-academic step. Selected students did not study at a faster rate than students who were rejected in the selection procedure, nor did they drop out less often than students who were rejected or refrained from (actively) participating in selection. In conclusion, we found only moderate support for a participation effect of selection, because selected students were more likely to complete their bachelor degree within three years than students who only opted for the lottery, but we observed no significant difference between selected students and students who withdrew from selection. Selected students did not outperform rejected students which suggests no selection effect.

These findings are in line with an increasing body of literature which has suggested that selection yields small gains in comparison to a weighted lottery [2, 17]. In the current study, selected and rejected students did not differ in terms of bachelor completion and dropout rates. However, as was previously suggested by Schripsema et al. [3, 4], participating in a selection procedure seems more predictive of study success than being successful in selection. It appears that those who refrain from (actively) participating in the selection procedure need more time to complete their courses and obtain their bachelor degree in comparison to those who actively participate in selection. The concurrent use of lottery and selection procedures may have instigated a self-selection mechanism in which students who are likely to underperform in the preclinical phase of medical study are less likely to participate in a demanding selection procedure. It is unclear why students withdraw from selection. Qualitative research may unravel the mechanism through which applicants decide to withdraw. It may reflect their doubts about their study choice. Doubt has been found to negatively affect students’ well-being and performance during medical study [23]. Participating in a selection procedure can stimulate an informed choice for studying medicine [24]. Self-selection is strongly influenced by students’ perceived chances of being successful in selection when admissions are only selection-based. An issue of concern is that applicants’ perceived chances of success are related to their background characteristics, especially their access to medical professionals in their network [25]. This is important to consider with respect to widening access to medical school. Further research on the experiences of students who refrain from active participation in selection is needed.

We did not find support for the previously suggested importance of an academic step for the selection of students who will perform well in the theoretical phase of the study [11, 18]. Applicants who experience less difficulty in performing well in pre-university education may be more inclined to invest time in substantial extracurricular activities, resulting in success in the selection procedure. This may explain why the academic selection step did not seem to add to the first selection step.

Several confounders, i.e., pu-GPA, age, gender and cohort, seemed to influence the association between the admission steps and the outcome variables. Students from cohorts 2008 and 2009 might have felt more pressure to graduate earlier due to governmental policy to introduce higher tuition rates in 2012 for students taking longer than four years to obtain a bachelor degree. An issue of concern is that students with certain background characteristics may be disadvantaged. Low socio-economic status, being a first-generation university student, ethnic minority background, having to provide care or contribute financially to family, and attending a public school may reduce students’ opportunities to obtain good secondary school grades and engage in extracurricular activities [26–29]. It may be that these groups of students withdraw or refrain from selection [25], yet their enrolment would contribute to the desired student diversity [30]. Moreover, the differences between the students who had and who had not participated seem to decrease throughout medical study. Significant differences between applicants who were selected and applicants who did not actively participate were most profound in the Year-1 outcome (40.4% versus 21.4%), and decreased after three (64.2% versus 42.9% with a bachelor degree) or four years (81.5% versus 74.3% with a bachelor degree). Previous research has indicated that performance differences cannot be ascribed to differences in motivation [10, 31]. Possibly, non-traditional students need more time to make the transition from pre-university to university education, as they have difficulty considering themselves fit for university [32]. This hypothesis could not be investigated, as legal restrictions impede the investigation of the demographics of the group of applicants who refrained from participating in selection.

We found no significant differences between the selected students and the students who were rejected after the non-academic step of the selection procedure. This may be due to the emphasis on theory and knowledge tests in the preclinical phase of the study. The non-academic elements assessed in the first step of the selection procedure can be expected to be more predictive of the interpersonal and intrapersonal aspects that are called upon in the clinical phase of medical study. Studies on older cohorts in the Netherlands, i.e., 2001–2004, using the same two-step selection procedure found that success in the non-academic step was related with better clerkship performance [15, 33]. Future research could reveal whether this relation exists among students in more recent cohorts.

### Implications

A two-step selection procedure, consisting of a non-academic and an academic step, seems effective for attracting students who are less likely to experience a delay in their studies. However, the practical relevance of the small, though statistically significant, differences is questionable. The financial benefit of admitting more students who go on to obtain their bachelor degree on time could be weighed against the costs of performing a selection procedure to attract these students and the risk of decreasing student diversity. At another medical school, a selection procedure was shown to be cost-beneficial in comparison with a lottery procedure [34]. As such comparisons are highly context-dependent, each medical school should perform an evaluation based on their own situation. In the long term, for some medical schools, it may be more cost-efficient to invest in improving education for the entire cohort than to invest in selecting students who are likely to perform the best. It should be noted that most students are able to complete the preclinical programme, i.e., few students drop out.

### Limitations

In the analyses, we were able to control for confounders such as age, gender, cohort and pu-GPA. However, medical school performance is also influenced by student characteristics, such as ethnic background and being a first-generation university student [12], for which we could not control. Another limitation is that we were unable to take into account the reasons for study delay, which may include lower (non-)academic ability, but also personal issues and undertaking valuable extracurricular activities, such as leadership roles in student organizations. Future research should include these additional variables. Due to legal restrictions, these data were unavailable for our research. The secondary research questions were of explorative nature; therefore, these results should be interpreted with caution. Furthermore, the groups of students who withdrew before step 1 as well as those who were rejected in step 2 were relatively small, which may explain why some findings did not reach significance.

## Conclusions

To date, there is no strong evidence for the added value of a qualitative selection procedure in comparison to a weighted lottery. The current study again lacks clear support. The study does, however, offer moderate support for the notion that the concurrent use of selection and lottery instigates a self-selection mechanism in which students who are likely to experience a study delay in the preclinical phase of medical study are less likely to participate in a demanding selection procedure. Students at risk for study delay seem likely to refrain from applying to a demanding selection procedure when a less demanding admission route is available. Future research should provide more insight into how the non-academic and academic selection steps contribute to the selection of students who will perform well in the clinical phase.

## Abbreviations

EC: European Credit
pu-GPA: pre-university grade point average
